# The Role of miR-144/Nrf2 Pathway in Muscle Oxidative Stress Induced by Oxidized Fish Oil in *Megalobrama amblycephala*, with an Emphasis on Protein Oxidation

**DOI:** 10.3390/antiox14101223

**Published:** 2025-10-11

**Authors:** Jie Yang, Xiaochuan Zheng, Qunlan Zhou, Changyou Song, Hongyan Tian, Aimin Wang, Xiangfei Li, Bo Liu, Cunxin Sun

**Affiliations:** 1 Wuxi Fisheries College, Nanjing Agricultural University, Wuxi 214081, China; 2 Key Laboratory of for Genetic Breeding of Aquatic Animals and Aquaculture Biology, Freshwater Fisheries Research Center, Chinese Academy of Fishery Science, Wuxi 214081, China; 3 Department of Ocean Technology, Yancheng Institute of Technology, Yancheng 224007, China

**Keywords:** oxidized fish oil, miR-144/Nrf2, antioxidant defense, protein oxidation, synthesis and hydrolysis, meat quality

## Abstract

This study investigated the role of *miR-144* in mitigating oxidized fish oil (OFO)-induced muscle oxidative stress and quality deterioration in *Megalobrama amblycephala*. The feeding trial was conducted for 5 weeks, and four experimental diets were formulated, namely NC (fresh fish oil), OF (OFO), OF + ago (OFO and *miR-144* agomir), and OF + anta (OFO and *miR-144* antagomir). Histological results showed that OFO significantly reduced myofiber density (from 758.00 ± 13.69 to 636.57 ± 13.44 N/mm^2^) and decreased the percentage of myofibers with diameters > 50 μm (from 53.45% to 38.52%). OFO intake significantly increased the content of malondialdehyde (MDA), protein carbonyl (PC), advanced oxidation protein product (AOPP), and 3-nitrotyrosine (3-NT), and significantly decreased superoxide dismutase (SOD) and glutathione peroxidase (GPx) activity in muscle. OFO treatment significantly up-regulated the expression of inflammatory factors (*NF-κB*, *TNF-α*, *HO-1*, and *IL-6*), significantly down-regulated *NQO1*. Moreover, OFO reduced muscle differentiation and maturation by down-regulating the expression of *MyoG*, *MYHC1*, and protein synthesis genes (*AKT3*, *TOR*, and *S6K1*), and up-regulating the expression of protein hydrolysis genes (*FoxO3a*, *MuRF1*, *HSP70*, *Beclin-1*, *P62*, and *ATG8*). Moreover, miR-144 agomir exacerbated OFO-induced muscle damage by suppressing *Nrf2*, whereas miR-144 antagomir mitigated these effects. Silencing miR-144 re-activates *Nrf2*, alleviating oxidative damage, enhancing protein deposition, and improving muscle quality. These findings suggest that targeting the miR-144/Nrf2 axis could counteract OFO-induced muscle deterioration.

## 1. Introduction

Fish oil is an indispensable fat component in aquatic feed, which is rich in fat-soluble vitamins and polyunsaturated fatty acids (PUFA), especially eicosapentaenoic acid (EPA) and docosahexaenoic acid (DHA) [1]. Studies have shown that fish oil is more effective than vegetable oil in enriching long-chain n-3 PUFA in animal muscles [2], and EPA and DHA in fish oil can reduce muscle protein oxidation in animals [3]. However, due to the chemical properties of polyunsaturated fatty acids, fish oil is prone to auto-oxidation. Under the action of heat, light, trace metals, or enzymes, auto-oxidation is enhanced, and lipid free radicals are formed, which react with oxygen to form lipid hydroperoxide as the main oxidation product [4]. A large amount of evidence shows that oxidized fish oil can cause severe oxidative stress and inflammatory responses in aquatic animals. What is more, it can lead to lipid peroxidation of cell membrane, destroy the integrity and function of cell membrane, and then affect enzyme activity and signal transduction in cells, resulting in tissue function damage, which may eventually lead to decreased meat quality [2,5,6,7].

Muscle is the primary part of human consumption and the largest protein library of the fish body. Its quality directly affects the acceptance of and nutritional value for consumers. Muscle texture characteristics can directly reflect muscle quality. The hardness of fish is positively correlated with good taste, and the antioxidant defense of fish is proportional to the relative shearing force [8,9]. And the low hardness and springiness of fish meat mean an increase in muscle lipids, which increases the fishy and oily flavor and affects the taste [10]. Oxidative lipid oxidation triggers oxidative stress, leading to the degradation of muscle proteins (such as myofibrillar proteins), which loosens the meat. Studies have shown that ingestion of oxidized fish oil can reduce fish muscle hardness, gumminess, and chewiness [1,11] and triggers oxidative stress [12]. Oxidative stress is one of the key factors in muscle quality decline. Proteins are the most vulnerable molecules to oxidative damage in cells [13], mainly because they are rich in easily oxidized amino acid residues, which are the targets of reactive oxygen species (ROS) oxidation [14]. Protein oxidation is a complex process, which can be attributed to the direct interaction between protein and ROS, or the direct interaction between protein and the secondary products of other oxidation processes such as lipid oxidation [15,16]. ROS, including superoxide anion (O_2_^−^), hydrogen peroxide (H_2_O_2_), and hydroxyl radicals (OH·) [17], as well as secondary products of lipid oxidation, such as malondialdehyde (MDA) and 4-hydroxynonenal (4-HNE) [18], are highly reactive molecules capable of attacking protein structures [19]. And these reactions will cause protein cross-linking and aggregation, resulting in increased carbonyl and disulfide bonds, reduced active sulfhydryl groups, and loss of protein solubility [20]. In addition, oxidative stress can also significantly affect the process of protein synthesis and hydrolysis, and even cause muscle dysplasia [21]. Oxidative stress inhibits protein synthesis by activating negative regulators (such as AMP-activated protein kinase, *AMPK*) or directly disrupting mechanistic targets of rapamycin (mTOR) signaling. At the same time, oxidative stress also activates the AKT–FoxO pathway, promotes the expression of muscle-specific E3 ubiquitin ligases (such as *MAFbx/Atrogin-1* and *MuRF1*), and accelerates protein degradation [22]. This imbalance between synthesis and degradation eventually leads to a net loss of muscle protein, which in turn affects muscle quality. So far, most of the studies on the effects of oxidized fish oil in fish have focused on the growth [23], antioxidant capacity [24], lipid oxidation [25], and inflammatory regulation [5,26] of aquatic animals. The effects of oxidized fish oil diet on fish muscle protein oxidation level, muscle cell differentiation and maturation, protein synthesis, and degradation metabolism have not been fully elucidated.

Moreover, intestinal flora can affect the deposition efficiency of fish muscle protein through a variety of mechanisms. On the one hand, intestinal flora is involved in the decomposition and metabolism of feed protein. The protease produced by intestinal flora can promote the hydrolysis of protein into small peptides and free amino acids, improving the utilization rate of feed protein by the host [27]. On the other hand, the metabolites (such as short-chain fatty acids, SCFAs) of specific probiotics (such as *Bacillus*) can exert anti-inflammatory properties and reduce the inflammatory response, thereby reducing the energy consumption of protein catabolism and promoting muscle protein synthesis [28,29]. In addition, SCFAs can regulate the proliferation and differentiation of myofibers by activating the mTOR signaling pathway [30]. However, the molecular mechanism of intestinal microbes affecting fish muscle development and protein deposition remains to be further elucidated.

Nuclear factor erythroid 2-related factor 2 (*Nrf2*) plays a central role in the cellular antioxidant defense system [31]. Under oxidative stress, the cysteine residue of Kelch-like ECH-associated protein 1 (Keap1) is modified by ROS, resulting in its conformational change, thereby releasing Nrf2. The free Nrf2 is transferred to the nucleus, forms a heterodimer with Maf protein, binds to the antioxidant response element (ARE), initiates the transcription of downstream antioxidant genes (such as *HO-1*, *NQO1*, *SOD*), and enhances the antioxidant capacity of cells [32], thereby protecting the body from DNA, protein, and/or lipid oxidative damage [33]. *Nrf2* is also regulated by miRNAs at the post-transcriptional level. miRNAs can directly regulate the expression of *Nrf2* by targeting its 3′-UTR, or indirectly affect the activation of *Nrf2* by targeting its upstream regulatory factors (such as *Keap1*) [34]. For example, up-regulation of *miR-200a* expression can inhibit *Keap1* and activate *Nrf2* signaling to inhibit dexamethasone-induced reactive oxygen species production in osteoblasts [35]. *MiR-216a* can directly regulate the expression of *Nrf2* by binding to the 3′-UTR of *Nrf2*, so inhibiting *miR-216a* can re-activate the expression of *Nrf2* to protect *Siniperca chuatsi* from cadmium-induced muscle oxidative stress [36]. Our team previously found that *Nrf2* is a target gene of *microRNA-144 (miR-144)* by in vitro luciferase reporter gene analysis [37]. And a study has found that down-regulation of *miR-144* increased the activation of *Nrf2* and alleviated cell injury and oxidative stress induced by ischemia/reperfusion [38]. In addition, studies have found that host or exogenous miRNA can directly or indirectly shape the intestinal flora. Xu et al. [39] directly proved that plant bol-*miR159* can be taken up by intestinal bacteria and regulate their growth by targeting specific bacterial genes (such as *ceIC*, *rnY*), thereby changing the overall intestinal flora structure of the host. Yan et al. [40] found that dietary chlorogenic acid specifically promoted the growth of probiotic *Lactobacillus johnsonii* by up-regulating the expression of host *miRNA-129-1-3p*, thereby improving intestinal inflammation. Therefore, we speculate that *miR-144* may affect inflammation by changing the intestinal environment of *M. amblycephala*. However, the regulatory role of miR-144/Nrf2 in oxidized fish-oil-induced meat quality decline and intestinal microbial imbalance has not been elucidated.

Therefore, the purpose of this study was to investigate the effects of oxidized fish oil diet on muscle inflammatory response and protein oxidation in *M. amblycephala* by quantitative analysis of enzyme activity and gene expression levels, and to evaluate the comprehensive effect of the *miR-144*/*Nrf2* pathway on muscle quality.

## 2. Materials and Methods

### 2.1. Ethics Statement and Experimental Design

The experimental procedures were conducted in accordance with the standards for scientific breeding and the utilization of fish established by the Animal Care and Use Committee of the Committee on the Ethics of Animal Experiments of the Freshwater Fisheries Research Center (LAECFFRC-2023-09-28).

Following a 2-week acclimation period, 120 vigorous *M. amblycephala* (initial body weight: 7.58 ± 0.09 g) were randomly assigned to four dietary and treatment regimens, each with three replicates (10 fish per replicate). The groups were as follows: a negative control (NC) fed a normal fish oil diet (CON), a positive control (OF) fed an oxidized fish oil diet (OFO), an OF + ago group fed OFO and injected with miR-144 agomir, and an OF + anta group fed OFO and injected with miR-144 antagomir. The feed formulation and approximate analysis are shown in Table 1.

To achieve the in vivo overexpression and inhibition of *miR-144*, a corresponding agomir and antagomir (GenePharma, Shanghai, China) were employed. The *miR-144* agomir’s sense strand sequence (5′-UACAGUAUAGAUGAUGUACU-3′) matched the mature miRNA sequence. Contrastingly, the *miR-144* antagomir’s sequence (5′-AGUACAUCAUCUAUACUGUA-3′) was the reverse complement. Both were reconstituted in DEPC-treated water (Biosharp, Hefei, China) at 0.75 nmol/μL. Based on pre-experiment results, fish in the OF + ago and OF + anta groups, after 1 week of receiving the oxidized fish oil diet, received weekly intraperitoneal injections of the respective oligonucleotide at 3 nmol·g^−1^ body mass for 35 days. Concurrently, the NC and OF groups were injected with an equal volume of DEPC-treated water.

### 2.2. Oxidized Fish Oil and Experimental Fish Management

The production method of oxidized fish oil was the same as that in a previous study [12]. The oxidized fish oil was prepared under laboratory conditions by adding Fe^2+^ (30 mg·kg^−1^, FeSO_4_·7H_2_O), Cu^2+^ (15 mg·kg^−1^, CuSO_4_·5H_2_O), H_2_O_2_ (600 mg·kg^−1^, 30%), and water (0.3%) in proportion to fresh fish oil. The mixture was thoroughly blended and then subjected to stirring and oxidation at 80 °C. The final peroxide value of oxidized fish oil was 323 mmol/kg.

During the breeding period, *M. amblycephala* were fed twice daily to apparent satiation with respective diets for 5 weeks, at 7:00–8:00, 17:00–18:00, respectively. To ensure sufficient oxygen, continuous aeration was conducted on a 24 h basis. In order to reduce human interference and prevent additional stress, the environment was kept as quiet as possible. The quality of the water was assessed every week. The feeding and death of *M. amblycephala* were observed daily. The water maintained an optimal range of 26~28 °C, with dissolved oxygen > 6.0 mg/L, ammonia and nitrite nitrogen content < 0.10 mg/L, and pH fluctuating between 6.5 and 7.0.

### 2.3. Sample Collection

After the 5-week breeding experiment, each tank of fish was weighed. Subsequently, all the fish in each group were fished out and added to 100 mg·L^−1^ MS-222 for rapid anesthesia. Meanwhile, 3 fish were taken from each tank, 9 fish in each group, and then muscle samples on the left side of the fish were quickly obtained on ice. The procured muscle tissues were rapidly cryopreserved with liquid nitrogen to impede degradation immediately and stored at a cryogenic temperature of −80 °C for quantifying the relative mRNA expression profiles in muscle tissue. Muscle samples from the other side of the same fish were taken and stored at −20 °C for biochemical analysis.

### 2.4. Muscle Texture Analysis

A total of 2 fish were selected from each tank, with 6 fish in each group. After removing the epidermis, a piece of meat (about 1 cm^3^) was taken from the middle of the right back muscle, and texture profile analysis (TPA) and shear force analysis of the muscle were performed using the Universal TA research texture analyzer (Tengba, Shanghai, China). The test conditions refer to the method of Ge et al. [41]. In short, shearing force was determined with a blade probe, compressing the sample to 50% of its thickness at 12 cm/min, and the maximum force (N) was recorded. Hardness was assessed via TPA using a cylindrical probe under identical compression and speed settings, with the peak force (g) from the first compression cycle taken as the value. Adhesiveness was determined after the first compression cycle as the area of the negative peak when the probe returned. Cohesiveness was calculated as the ratio of the work area of the second compression to the work area of the first compression. Springiness was assessed as the height of the sample recovered during the interval between two compressions. Among then, Gumminess = Hardness × Cohesiveness, Chewiness = Hardness × Cohesiveness × Springiness.

### 2.5. Histopathology of Muscle

After 48 h fixation in 4% paraformaldehyde (Biosharp, Hefei, China), muscle samples were processed according to the standard procedures and cut into approximately 4 μm sections. After unfolding and drying, the sections were stained with hematoxylin and eosin (H&E) (Biosharp, Hefei, China) and examined under an optical microscope (Olympus BX51, Tokyo, Japan).

### 2.6. Antioxidant Enzyme Activity and Evaluation of Protein Oxidation Levels

About 0.1 g of muscle tissue was taken, added with 1 mL extract, homogenized at 4 °C, and centrifuged at 4 °C, 12,000 rpm for 10 min. Antioxidant enzyme activities (SOD and GPx) and MDA content in the supernatant were determined by commercial kits (Jiangsu Aidisheng Biological Technology Co., Ltd., Yancheng, China). Protein oxidation indexes PC, AOPP, and 3-NT were determined by Enzyme-Linked Immunosorbent Assay (ELISA) kits (Jiangsu Aidisheng Biological Technology Co., Ltd.). All steps are carried out in strict accordance with the instructions.

### 2.7. Quantitative Real-Time PCR

RNAiso Plus (Takara, Dalian, China) was utilized to extract total RNA from muscle tissues of four experimental groups (9 samples in each group). The RNA concentration was determined by measuring the absorbance at a wavelength of 260:280 nm (OD260/OD280 = 1.8–2.0) using a Nanodrop 2000 (Thermo Fisher Scientific, Massachusetts, USA). First-strand cDNA was synthesized for qRT-PCR analysis using the HiScript^®^ III RT SuperMix (+gDNA wiper) (Vazyme, Nanjing, China). qRT-PCR was conducted with TB Green^®^ Premix Ex Taq™ II (Tli RNaseH Plus) (Takara, Dalian, China), in accordance with the manufacturer’s instructions. *β-actin* and *5S RNA* served as the reference genes for mRNA and miRNA, respectively, and three technical replicates were tested for each sample. Online design tools (NCBI, Bethesda, MA, USA) were employed for primer design (Table 2). The CDS sequence for designing the selected gene primers was obtained from the transcriptome sequencing database of *M. amblycephala* muscle tissue in our laboratory. The results were calculated using the 2^−ΔΔCt^ method, and the gene-specific primers were synthesized by Shanghai Generay Biotech Co., Ltd. (Shanghai, China).

### 2.8. 16S rRNA Sequencing Analysis

The collected intestinal chyme samples were used for 16S rRNA high-throughput sequencing of intestinal microbiota. In short, tissue DNA was extracted using the NucleoSpin Soil kit (Macherey-Nagel, Germany). Subsequently, the universal primers (341F: 5′-CCTACGGGNGGCWGCAG-3′, 806R: 5′-GGACTACHVGGGTWTCTAAT-3′) of the V3-V4 region of the 16S rRNA gene were used for PCR amplification. The PCR products were detected by 2% agarose gel electrophoresis and purified using the AxyPrep DNA Gel Extraction Kit (Axygen Biosciences, CA, USA). Finally, the purified products were subjected to library construction and sequencing analysis by Nanjing Genepioneer Biotechnology Co., Ltd. (Nanjing, China).

### 2.9. Statistical Analysis

All numerical results were expressed as mean ± standard error of the mean (X ± SEM). One-way ANOVA was performed using SPSS 24.0 to compare differences among groups. A *p*-value < 0.05 was considered statistically significant. Prior to statistical analysis, the assumption of normal distribution and homogeneity of variances was confirmed for all datasets. Tables were formatted using Excel 2016, while graphical representations were generated using GraphPad Prism, version 10.1.

## 3. Results

### 3.1. Transcription Levels of miR-144, Nrf2, and Keap1 Genes

**Figure 1:**
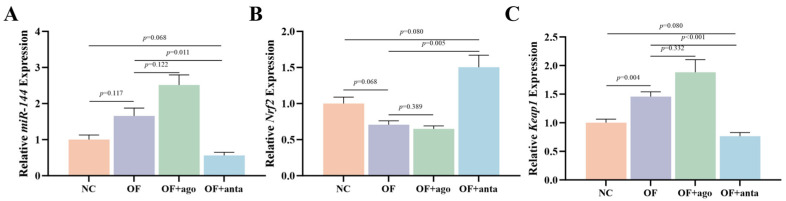
The transcription of *miR-144* (**A**), *Nrf2* (**B**), and *Keap1* (**C**) genes in muscle of *M. amblycephala*. The results are expressed as the mean ± SEM, *n* = 9. miR-144, microRNA-144; Nrf2, nuclear factor-erythroid 2-related factor 2; Keap1, Kelch-like ECH-associated protein 1.

As shown in Figure 1, compared with the NC group, oxidized fish oil significantly increased the expression of *Keap1* (*p* < 0.01) in muscle. After injection of miR-144 antagomir, compared with the OF group, the relative expression of *miR-144* and *Keap1* was significantly down-regulated (*p* < 0.05). Conversely, compared with the OF group, the expression of *Nrf2* in the OF + anta group was significantly up-regulated (*p* < 0.01).

### 3.2. Effects of Oxidized Fish Oil and miR-144 Interference on Muscle Texture Characteristics of M. amblycephala

**Figure 2:**
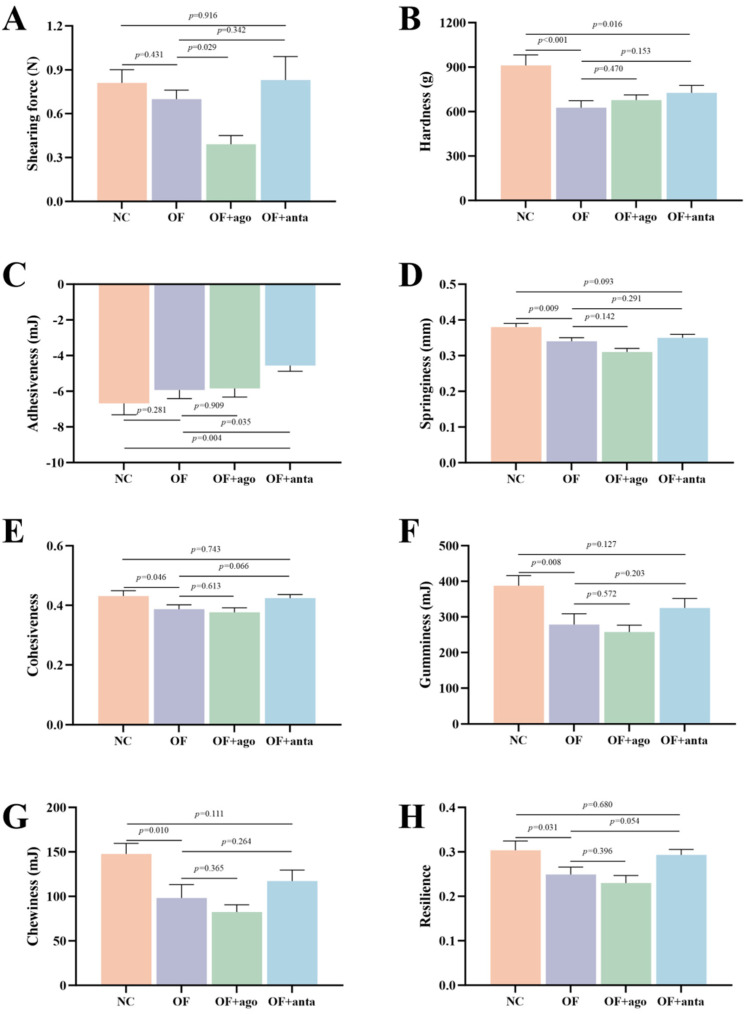
Effects of oxidized fish oil and *miR-144* interference on muscle texture characteristics of *M. amblycephala*. (**A**) Shearing force; (**B**) Hardness; (**C**) Adhesiveness; (**D**) Springiness; (**E**) Cohesiveness; (**F**) Gumminess; (**G**) Chewiness; (**H**) Resilience. The results are expressed as the mean ± SEM, *n* = 6.

As shown in Figure 2 and Table S1, oxidized fish oil significantly reduced the muscle hardness, springiness, cohesiveness, gumminess, chewiness, and resilience of *M. amblycephala* (*p* < 0.05). After injection of miR-144 agomir, compared with the OF group, the shearing force was significantly reduced (*p* < 0.05), and other texture characteristics were reduced, but there was no statistical difference. After inhibiting *miR-144*, compared with the OF group, muscle springiness, cohesiveness, gumminess, chewiness, and resilience were increased to the level of the NC group.

### 3.3. Muscle H&E Staining

**Figure 3:**
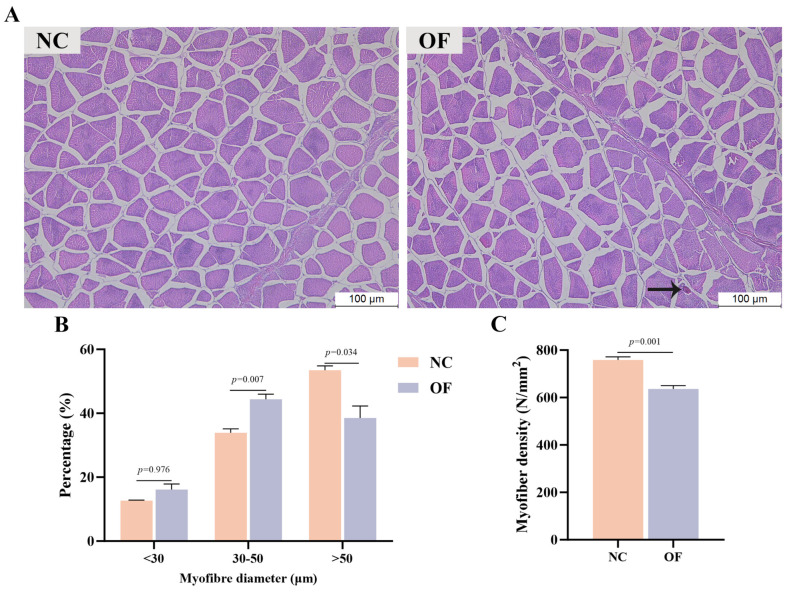
Effects of oxidized fish oil on muscle morphology (200×) (**A**), myofiber diameter frequency (**B**), and myofiber density (**C**) of *M. amblycephala*. Note: Black arrow shows the rimmed vacuole in muscle fiber. The results are expressed as the mean ± SEM, *n* = 6.

Figure 3 shows the H&E staining of muscle tissue sections of *M. amblycephala*. Histomorphology showed that after oxidized fish oil treatment, the myofiber gap of *M. amblycephala* increased, myofiber density was significantly reduced (*p* < 0.01), and some myofibers showed vacuolar degeneration. Compared with the NC group, the percentage of myofibers with a diameter of 30–50 μm in the OF group was highly significantly increased (*p* < 0.01), accompanied by a significant decrease in the percentage of myofibers with a diameter of more than 50 μm (*p* < 0.05).

### 3.4. Muscle Antioxidant Enzyme Activity and Protein Oxidation Level

**Figure 4:**
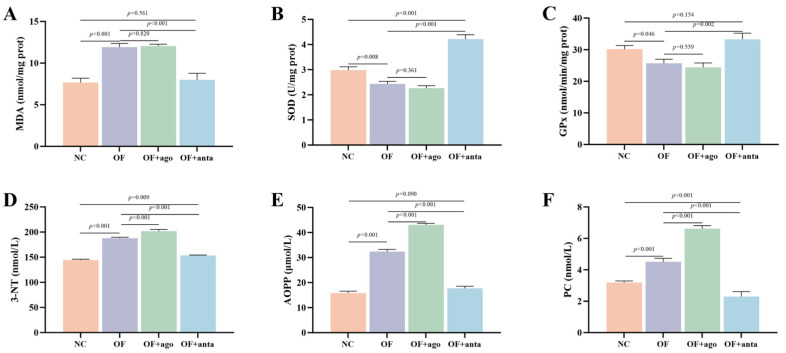
Effects of oxidized fish oil and *miR-144* interference on antioxidant enzyme activity and protein oxidation product level in muscle of *M. amblycephala*. The results are expressed as the mean ± SEM, *n* = 6. (**A**) MDA, malondialdehyde; (**B**) SOD, superoxide dismutase; (**C**) GPx, glutathione peroxidase; (**D**) 3-NT, 3-nitrotyrosine; (**E**) AOPP, advanced oxidation protein products; (**F**) PC, protein carbonyl.

As shown in Figure 4A, compared with the NC group, oxidized fish oil significantly increased the MDA content in muscle (*p* < 0.001). MDA content remained at a high level after injection of miR-144 agomir. After injection of miR-144 antagomir, the MDA content in the OF + anta group was significantly lower than that in the OF group (*p* < 0.001), which was reduced to the level of the NC group. In addition, as shown in Figure 4B,C, compared with the control group, muscle SOD (*p* < 0.01) and GPx (*p* < 0.05) activities in the OF group were significantly decreased. The activities of SOD (*p* < 0.001) and GPx (*p* < 0.01) in muscle of the OF + anta group were significantly higher than that of the OF group, and the activity of SOD was significantly higher than that of the NC group (*p* < 0.001).

Protein markers are shown in Figure 4B–D. As for the level of protein oxidation, the contents of PC, AOPP, and 3-NT in the OF group were significantly higher than those in the NC group (*p* < 0.001). After miR-144 agomir treatment, 3-NT, PC, and AOPP further increased (*p* < 0.001). After injection of miR-144 antagomir, compared with the OF group, these three indicators were significantly reduced (*p* < 0.001), and some were reduced to the level of the NC group.

### 3.5. Effects of Oxidized Fish Oil and miR-144 Interference on Inflammation-Related Genes in M. amblycephala Muscle

**Figure 5:**
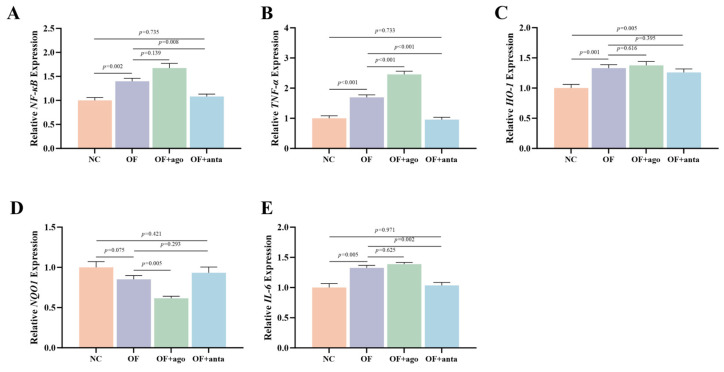
Effects of oxidized fish oil and *miR-144* interference on inflammation-related genes in muscle of *M. amblycephala*. The results are expressed as the mean ± SEM, *n* = 9. (**A**) NF-κB, nuclear factor kappa-B; (**B**) TNF-α, tumor necrosis factor α; (**C**) HO-1, heme oxygenase-1; (**D**) NQO1, NAD(P)H: quinone oxidoreductase 1; (**E**) IL-6, interleukin-6.

As shown in Figure 5, *NF-κB* (*p* < 0.01), *TNF-α* (*p* < 0.001), *HO-1* (*p* < 0.01), and *IL-6* (*p* < 0.01) were significantly up-regulated in the OF group, while *NQO1* showed a downward trend, but there was no statistical difference. In the OF + ago group, miR-144 agomir significantly up-regulated the expression of *TNF-α* (*p* < 0.001), and significantly down-regulated the expression of *NQO1* (*p* < 0.01). The expression of these genes was reversed by miR-144 antagomir, and *NF-κB* (*p* < 0.01), *TNF-α* (*p* < 0.001) and *IL-6* (*p* < 0.01) were significantly decreased compared with the OF group; only *HO-1* was still highly expressed and significantly higher than the NC group (*p* < 0.01).

### 3.6. Growth and Differentiation of Muscle Cells and Protein Synthesis and Hydrolysis

**Figure 6:**
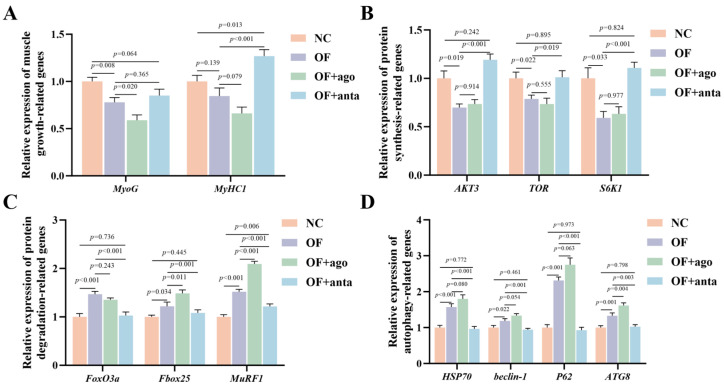
Effects of oxidized fish oil and *miR-144* interference on genes related to protein synthesis, hydrolysis, and muscle development of *M. amblycephala*: (**A**) muscle growth-related genes, (**B**) protein synthesis-related genes, (**C**) protein hydrolysis-related genes, (**D**) autophagy-related gene. The results are expressed as the mean ± SEM, *n* = 9. MyoG, myogenin; MyHC1, myosin heavy chain 1; AKT3, Ak strain transforming 3; TOR, target of rapamycin; S6K1, ribosomal protein S6 kinase Beta-1; FoxO3a, Forkhead box O3a; Fbox25, F-box protein 25; MuRF1, muscle RING finger 1; HSP70, heat shock protein 70; ATG8, autophagy-related protein 8.

As shown in Figure 6, compared with the NC group, oxidized fish oil treatment significantly down-regulated the expression levels of muscle cell growth and differentiation genes *MyoG* (*p* < 0.01) and muscle protein synthesis genes *AKT3* (*p* < 0.05), *TOR* (*p* < 0.05), and *S6K1* (*p* < 0.05). Meanwhile, the expression levels of muscle protein degradation genes *Fbox25* (*p* < 0.05), *FoxO3a*, and *MuRF1* (*p* < 0.001) were significantly up-regulated. In the OF + ago group, the expression of these genes showed a more unfavorable trend. Among them, *MyoG* (*p* < 0.05) decreased significantly, while *Fbox25* (*p* < 0.05) and *MuRF1* (*p* < 0.001) increased significantly. MiR-144 antagomir treatment significantly reversed the expression levels of these genes. The results showed that oxidized fish oil significantly increased the gene levels of *HSP70* (*p* < 0.001), *Beclin-1* (*p* < 0.05), *P62* (*p* < 0.001), and *ATG8* (*p* < 0.01) in muscle. After injection of miR-144 agomir, the expression of these three genes was further increased, but only *ATG8* was statistically different (*p* < 0.01). After miR-144 antagomir treatment, *HSP70* (*p* < 0.001), *Beclin-1* (*p* < 0.001), *P62* (*p* < 0.001), and *ATG8* (*p* < 0.01) were significantly reduced to normal levels.

### 3.7. Intestinal Microbiota Composition and Differences Analysis

**Figure 7:**
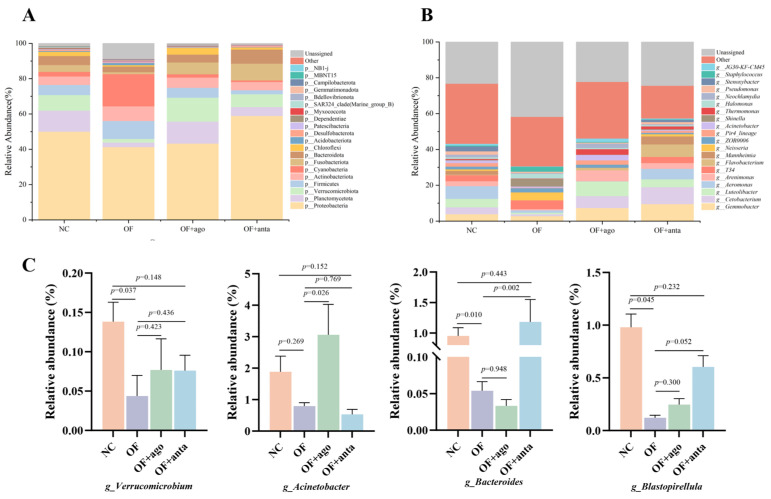
The effects and differences of oxidized fish oil and miR-144 interference on the composition of intestinal microbial community in *M. amblycephala*: (**A**) phylum level, (**B**,**C**) genus level.

As shown in Figure 7, the relative abundance of the top 20 microbiomes at the phylum level (Figure 7A) and genus level (Figure 7B) of the intestinal flora of each group was statistically analyzed. At the phylum level, Proteobacteria, Planctomycetota, Verrucomicrobiota, Firmicutes, and Bacteroidota were the main phyla. Compared with the NC group, the relative abundance of Proteobacteria, Planctomycetota, Verrucomicrobiota, and Bacteroidota in the OF group decreased, and Firmicutes increased, but there was no statistical difference. Compared with the OF group, the relative abundance of Firmicutes decreased after injection of miR-144 agomir. After injection of miR-144 antagomir, the relative abundance of Proteobacteria, Planctomycetota, Verrucomicrobiota, and Bacteroidota increased.

At the genus level, *Aeromonas*, *Luteolibacter*, *Gemmobacter*, *Cetobacterium*, and *Arenimonas* were the main genera. Compared with the NC group, the relative abundance of these genera in the OF group decreased. Compared with the OF group, the relative abundance of *Luteolibacter* and *Gemmobacter* in the OF + ago group increased significantly, while the relative abundance of *Aeromonas* and *Cetobacterium* decreased. In the OF + anta group, the relative abundance of *Gemmobacter*, *Cetobacterium*, and *Arenimonas* increased, while the relative abundance of *Aeromonas* decreased slightly.

As shown in Figure 7C, the relative abundance of *Verrucomicrobium*, *Acinetobacter*, *Bacteroides*, and *Blastopirellula* was analyzed. Compared with the NC group, the relative abundance of *Verrucomicrobia*, *Bacteroides*, and *Blastopirellula* (*p* < 0.05) in the intestine of *M. amblycephala* was significantly reduced by feeding oxidized fish oil. MiR-144 agomir further reduced the relative abundance of *Bacteroides*, but there was no statistical difference compared with the OF group. After injection of miR-144 antagomir, the relative abundance of *Bacteroides* (*p* < 0.01) was significantly reversed.

### 3.8. Correlation Analysis

**Figure 8:**
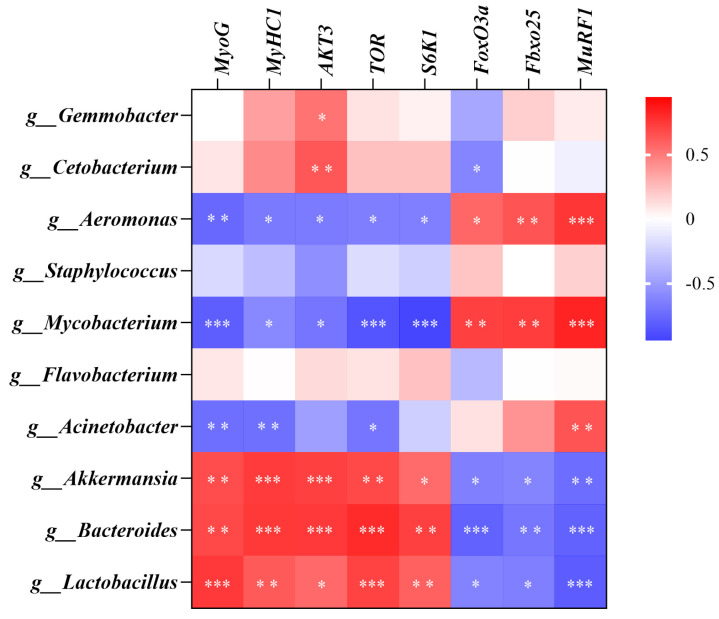
Correlation analysis between microorganisms and muscle fiber growth, protein synthesis, and degradation-related genes. * represents *p* < 0.05, ** represents *p* < 0.01, *** represents *p* < 0.001.

Figure 8 shows the Pearson correlation analysis between mRNA levels of muscle development, protein synthesis, and metabolism-related genes and intestinal microbes. The results showed that *Aeromonas*, *Mycobacterium*, and *Acinetobacter* were negatively correlated with the expression of genes related to muscle development and protein synthesis but positively correlated with genes related to protein degradation. *Akkermansia*, *Bacteroides*, and *Lactobacillus* were positively correlated with the expression of genes related to muscle development and protein synthesis but negatively correlated with genes related to protein degradation.

## 4. Discussion

A large number of studies have shown that oxidized fish oil feed will adversely affect the physiology of aquatic animals. For example, long-term feeding of oxidized fish oil causes decreased growth performance [48], liver oxidative stress [49], enteritis [26,50], and decreased muscle quality [23]. In view of the high market economy value of muscle in aquatic animals, it is of great significance to explore the mechanism of oxidized fish oil damaging muscle quality and screen key intervention targets. Thus, we explored the effects of oxidized fish oil on muscle antioxidant and meat quality of *M. amblycephala* and focused on the effects of oxidized fish oil on muscle protein oxidation, protein synthesis, and hydrolysis, and the regulatory role of the miR-144/Nrf2 pathway.

Higher muscle hardness and springiness mean better taste. The protein in meat can form a network structure with its hydration layer, and has a certain ability to resist external forces, which is manifested as the springiness of meat. Cohesiveness reflects the ability of food to resist damage when chewing food and is closely connected to keeping food intact [51]. Studies have confirmed that muscle hardness and shearing force are positively correlated with myofiber diameter and density [52,53], and springiness is negatively correlated with the degree of proteolysis [54]. This study found that oxidized fish oil caused a decrease in muscle hardness, springiness, and gumminess, reduced chewing experience, and ultimately caused a decrease in overall taste. Moreover, a positive correlation exists between muscle hardness and myofiber density [55]. This study found that the addition of oxidized fish oil to the diet increased the proportion of muscle fibers to a diameter of 30–50 μm but reduced the proportion of myofibers to a diameter greater than 50 μm. And from the results of histomorphology, oxidized fish oil increased the gap between myofibers, and the number of myofibers per unit area was significantly reduced, and even vacuolar degeneration occurred in myofibers. This is consistent with the texture results. Similar results were found in the study of *Piaractus mesopotamicus* by Salomó et al. [56] and hybrid striped bass by Li et al. [57]. The higher percentage of myofibers with a diameter ≤20 μm indicates fiber hyperplasia, and the higher percentage of myofibers with a diameter >50 μm indicates fiber hypertrophy [58]. This also indicates that muscle hypertrophy in the OF group is inhibited, which is presumably caused by oxidized fish-oil-induced oxidative stress. Therefore, it can be determined that oxidized fish oil inhibits myofiber growth and may further affect meat quality.

The growth of myofibers depends on a process called myogenesis, which plays a key role in normal muscle development and regeneration after injury [59]. Myogenesis is regulated by a variety of muscle regulatory factors [60,61]. Thus, the gene expression of *MyoG* and *MyHC1*, which are closely related to muscle development and differentiation, was detected in this experiment. The results showed that oxidized fish oil down-regulated the relative expression of *MyoG* and *MyHC1*. *MyoG*, as the main regulatory factor in the process of myogenesis, is an essential regulatory factor for the differentiation of muscle precursor cells into mature muscle fibers [62]. Its expression initiation marks the beginning of the differentiation process of muscle cells. *MyHC1* is one of the key proteins in muscle contraction, and its expression is on the rise in the early stage of muscle cell differentiation [63]. In addition, during the differentiation and maturation of muscle cells, *MyoG* can directly or indirectly regulate the expression of the *MyHC1* gene, making it express in the same trend as it [64]. Combined with these views, down-regulation of *MyoG* and *MyHC1* expression usually means impaired muscle differentiation and regeneration and may break the balance between muscle protein synthesis and degradation and reduce muscle quality. In this study, overexpression of *miR-144* further inhibited the expression of *MyoG* and inhibited myofiber growth. Silencing the expression of *miR-144* activated the expression of *MyoG* and *MyHC1*, which promoted the differentiation and maturation of muscle cells. This is similar to the results of Motohashi et al. [65], who found that inhibition of *miR-128a* promoted muscle cell proliferation and myotube hypertrophy.

Studies have shown that muscle growth and protein metabolism are closely related to the mTOR pathway [66]. *mTOR* can promote a variety of cellular anabolism. *mTORC1* promotes protein synthesis by phosphorylating *S6K1* and *4E-BP1* [21]. *mTORC2* can activate *AKT* by phosphorylation at the Ser473 site of *AKT*. Activated *AKT* promotes *Rheb* activation of *mTORC1* by inhibiting the TSC1/2 complex, thereby enhancing protein synthesis [67]. In addition, *AKT* can also reduce catabolism by inhibiting autophagy [68]. In this study, oxidized fish oil significantly reduced the gene levels of *AKT3*, *TOR*, and *S6K1* in muscle, which was similar to the results of the study of oxidative stress induced by aflatoxin B1 on the muscle damage of grass carp in He et al. [69], indicating that oxidized fish oil causes muscle damage in *M. amblycephala*. However, the maintenance of muscle mass is not only dependent on protein synthesis but also strictly regulated by protein degradation. Protein degradation is mainly achieved through the ubiquitin-proteasome system (UPS) and autophagy–lysosomal pathways [70]. *MuRF1* and *Fbxo25* (the F-box protein homolog of atrophin-1) are the key components of the UPS [71,72], while HSP70, *Beclin-1*, *P62*, and *ATG8* are the core regulatory genes of the autophagy–lysosomal pathway. At the same time, *FoxO3a* plays an important regulatory role in protein degradation by coordinating the UPS and the autophagy–lysosomal pathway. The results of this study showed that oxidized fish oil significantly up-regulated the expression of these genes, which was similar to the results of Tacchi et al. [73], indicating that oxidized fish oil activated the UPS and autophagy–lysosomal pathways and promoted protein degradation. It is worth noting that in this study, while the autophagy of the OF group was enhanced, the gene level of *P62* also increased significantly, which was the same as our previous experimental result [12]. It is speculated that excessive oxidative stress interferes with the *P62*-mediated autophagy flux and hinders the degradation of *P62*. And it was found that overexpression of *miR-144* did not further significantly inhibit the protein synthesis pathway but aggravated the protein autophagy and UPS degradation pathway. Silencing *miR-144* can significantly promote muscle protein synthesis and inhibit its degradation.

This study also observed that oxidized fish oil affected the antioxidant system of *M. amblycephala* muscle, which was manifested by an increase in MDA content in muscle and a decrease in the activity of antioxidant enzymes SOD and GPx. This is similar to the results for *Eriocheir Sinensis* [74]. MDA is one of the final products of intracellular polyunsaturated fatty acid peroxidation, and an increase in free radicals will lead to its excessive production [75]. SOD and GPx are important antioxidant enzymes in the body. SOD converts superoxide free radicals into hydrogen peroxide, while GPx further reduces hydrogen peroxide to water, jointly preventing the excessive content of destructive free radicals in the body [76]. In addition, inflammatory and antioxidant genes *NF-κB*, *TNF-α*, *HO-1*, *IL-6*, and *HSP70* were up-regulated, while *Nrf2* and *NQO1* were down-regulated, which indicated that an inflammatory response had occurred in the muscle tissue of *M. amblycephala*. The interaction between oxidative stress and inflammatory response may further aggravate muscle damage. Activation of *NF-κB*, a key regulator of inflammatory response, not only promotes the expression of pro-inflammatory cytokines (such as *TNF-α* and *IL-6*) [77], but also may weaken the antioxidant defense ability of cells by inhibiting the *Nrf2* signaling pathway. *Nrf2* is the core transcription factor of the intracellular antioxidant response. When oxidative stress occurs, it dissociates with *Keap1* and translocates to the nucleus, and then binds to antioxidant response element (ARE) to regulate the expression of downstream antioxidant genes (such as *HO-1*, *NQO1*) [78]. In this study, *Nrf2* and *NQO1* were down-regulated synchronously, which further weakened the ability of cells to scavenge free radicals. However, *HO-1* increased with the inhibition of *Nrf2*, suggesting that other factors compensatory up-regulated the expression of *HO-1*. In summary, oxidized fish oil causes muscle tissue damage in *M. amblycephala* by inducing an inflammatory response and an imbalance of the antioxidant system. Overexpression of *miR-144* aggravated muscle inflammation by further inhibiting the expression of *Nrf2*. Inhibition of *miR-144* reactivated *Nrf2* expression, increased muscle antioxidant enzyme activity, and inhibited the expression of pro-inflammatory factors. It is indicated that *miR-144* can target *Nrf2* to regulate oxidative stress and inflammation in muscle induced by oxidized fish oil, which is similar to the results of Song et al. [37].

The effects of oxidized fish oil on protein oxidation were further analyzed. Protein oxidation is one of the important manifestations of oxidative stress, which is usually evaluated by markers such as PC, AOPP, and 3-NT. PC is a common marker of protein oxidative damage, formed through the oxidative deamination of basic amino acids under the attack of free radicals, representing an irreversible form of oxidative damage [79]. AOPP is the final oxidation product of proteins in the process of oxidative stress. Its high molecular structure is rich in dityrosine and carbonyl, which are closely related to oxygen free radical damage and oxidative stress in vivo [80]. In plasma, the generated AOPP induces more ROS production and aggravates oxidative stress by causing respiratory bursts of monocytes and neutrophils [81]. In other tissues, AOPP induces an inflammatory response through NADPH oxidase-dependent activation of *NF-κB* [82]. 3-NT is a product of nitration modification of tyrosine residues in proteins under the action of active nitrogen species such as peroxynitrite. The increase in its content symbolizes the aggravation of nitration stress damage [83]. In this study, oxidized fish oil significantly increased the content of PC, AOPP, and 3-NT in the muscle of *M. amblycephala*, indicating that the oxidative damage of protein was aggravated. This means that oxidative stress induced by oxidized fish oil leads to excessive production of free radicals, which attack protein molecules and destroy their structure and function. Protein damage will hinder the growth of myofiber and reduce myofiber density [84]. In this study, overexpression of miR-144 further aggravated muscle protein oxidation induced by oxidized fish oil, and inhibition of miR-144 alleviated it to a certain extent.

Recently, the important role of intestinal microbiota in the nutrition, immunity, and defense functions of animal hosts has attracted wide attention [85], and its community stability is crucial to host health. A study has shown that the addition of oxidized fish oil to feed may cause an imbalance in intestinal flora [86]. In this study, Proteobacteria were dominant in the intestinal microbiota of *M. amblycephala*, followed by Planctomycetota, Verrucomicrobiota, Firmicutes, and Actinobacteriota. After feeding oxidized fish oil, the abundance of *Proteobacteria* in the intestine of *M. amblycephala* decreased, while the abundance of Firmicutes increased, which was consistent with the results of Liu et al. [87] and Yu et al. [86]. This indicated that oxidized fish oil may lead to an imbalance in intestinal flora in *M. amblycephala*. It was found that oxidized fish oil significantly reduced the relative abundance of *Verrucomicrobium*, *Bacteroides*, and *Blastopirellula*. *Acinetobacter* and *Bacteroides* are the main bacteria of fish. *Acinetobacter* can help the host digest protein in carnivorous fish, while *Bacteroides* can help herbivorous fish digest cellulose [88]. Therefore, the decrease in the relative abundance of *Acinetobacter* and *Bacteroides* means that the digestion and absorption function of fish is reduced, reducing the energy supply required for protein deposition. Compared with the OF group, inhibition of *miR-144* significantly up-regulated the relative abundance of *Bacteroides* and was higher than that of the NC group. In conclusion, oxidized fish oil down-regulates the relative abundance of probiotics in intestinal, destroys the intestinal barrier function, and reduces the intestinal digestion and absorption capacity. Inhibition of *miR-144* may increase the abundance of probiotics, restore the microbial barrier, and reduce the inflammatory response. This is similar to the study of Xu et al. [39] in *miR-159*. miRNA can target bacterial genes and enter bacterial cells, indicating that miRNA may serve as a strategy to optimize host health by regulating intestinal microbial communities.

In order to further explore the relationship between intestinal microorganisms and muscle fiber growth, protein synthesis and decomposition, correlation analysis was performed. The results showed that *Akkermansia*, *Bacteroides*, and *Lactobacillus* were positively correlated with the expression of genes related to muscle development and protein synthesis but negatively correlated with genes related to protein degradation. Based on the existing research evidence, probiotics may promote the balance of muscle protein metabolism through a dual regulatory mechanism: on the one hand, they can up-regulate the protein synthesis pathway by activating the mTOR signaling pathway [89]. On the other hand, specific strains (such as *Lactobacillus*) can significantly inhibit the gene expression of *MuRF1* and *MAFbx*, thereby inhibiting the protein decomposition pathway of the ubiquitin-proteasome system and promoting protein deposition [90]. In addition, SCFAs produced by *Akkermansia*, *Bacteroides*, and *Lactobacillus* metabolism can affect the host’s energy metabolism and immune response, indirectly affecting muscle development and protein metabolism. For example, butyric acid can promote the growth and differentiation of muscle cells and inhibit muscle proteolysis [91]. Therefore, we hypothesized that the microbiota signatures observed in our study might be associated with improved muscle protein metabolism, potentially through similar mechanisms. However, whether miRNAs indirectly affect muscle development by affecting intestinal microbiota remains unclear and needs further study.

Numerous studies have demonstrated that miRNAs can serve as novel therapeutic targets for oxidative-stress-related complications and act as upstream regulators of the *Nrf2* signaling pathway, modulating its activity at multiple levels [35,92,93]. Our previous study confirmed that *miR-144* can inhibit *Nrf2* expression by binding to the 3′-UTR of *Nrf2* [37]. In view of this, in this study, *miR-144* was interfered by injection of *miR-144* agomir and *miR-144* antagomir to explore the regulatory role of *miR-144* in oxidative fish-oil-mediated muscle oxidative stress and protein damage in *M. amblycephala*. The results showed that *miR-144* agomir inhibited *Nrf2* expression, which mediated the up-regulation of *NF-κB* and *TNF-α* and the down-regulation of *NQO1*, and ultimately aggravated muscle inflammation induced by oxidized fish oil. Inhibition of *miR-144* significantly increased the expression of *Nrf2*, accompanied by an increase in antioxidant enzyme SOD and GPx activity, which effectively improved the antioxidant capacity of *M. amblycephala*, which was similar to the results of Li et al. [94]. In addition, *Nrf2* deficiency inhibits the PI3K-AKT-mTOR pathway [95] and activates autophagy, which reduces protein synthesis and further increases the UPS hydrolysis pathway of proteins. This experiment confirmed that a reduction in ROS in *M. amblycephala* by inhibiting *miR-144* to re-activate *Nrf2*, thereby alleviating muscle damage and inhibiting protein oxidation and hydrolysis. Not only that, the inhibition of *miR-144* also increased the expression of muscle differentiation and maturation factors and protein synthesis factors, which was conducive to muscle fiber development and ultimately improved meat quality.

## 5. Conclusions

In conclusion, oxidized fish oil impaired muscle quality in *M. amblycephala* by inducing inflammation, protein oxidation, and disrupting muscle protein synthesis and hydrolysis, along with altering intestinal microbiota. This study showed that inhibition of miR-144 can reduce muscle inflammation and protein oxidation induced by oxidized fish oil by activating the Nrf2 signaling pathway, regulating myogenic regulatory factors, and promoting muscle growth, differentiation, and protein synthesis. These results indicate that interfering with miR-144 provides new therapeutic strategies and molecular targets for the prevention and treatment of muscle dysplasia and related diseases. This study preliminarily revealed the key role of miR-144 in muscle oxidative stress of *M. amblycephala* and observed the correlation changes of intestinal flora. However, whether miR-144 indirectly affects muscle development by regulating intestinal microbiota remains an urgent problem to be solved. Future studies can analyze the effects and mechanisms of miR-144 on intestinal microbial metabolic functions, pathways, and products through metagenomics and identify metabolites related to intestinal physiological regulation of *M. amblycephala* to clarify the mechanism by which miR-144 regulates intestinal microbes and thus affects the physiology of aquatic animals.

## Figures and Tables

**Table 1:** Feed composition and basic nutrition level (%).

Ingredient (%)	CON	OFO
Fish meal	4	4
Cottonseed meal	17	17
Soybean meal	30	30
Rapeseed meal	15	15
Rice bran	2	2
Fish oil	6	0
Oxidized fish oil	0	6
α-starch	16	16
Calcium biphosphate	2	2
Vitamin premix ^1^	0.1	0.1
Mineral premixes ^2^	0.3	0.3
Carboxymethyl cellulose	3.1	3.1
Zeolite powder	4	4
Choline chloride	0.5	0.5
Total	100	100
Proximate analysis (%)		
Crude protein	31.74	31.79
Crude lipid	7.36	7.22
Gross energy (MJ/kg)	12.44	12.55

^1^ Vitamin contents per kg diets: Vitamin A, 900 IU; Vitamin B_1_, 0.32 mg; Vitamin B_2_, 1.09 mg; Vitamin B_5_, 2 mg; Vitamin B_6_, 0.50 mg; Vitamin B_12_, 0.0016 mg; Vitamin C, 5 mg; Vitamin D, 200 IU; Vitamin E, 4.50 mg; Vitamin K_3_, 0.22 mg; Niacin, 2.80 mg; Folic acid, 0.17 mg; Pantothenate, 1 mg; Choline, 60 mg; ^2^ Mineral contents per kg diets: FeSO_4_⋅7H_2_O, 75 mg; CuSO_4_⋅5H_2_O, 6 mg; ZnSO_4_⋅7H_2_O, 66 mg; Na_2_SeO_3_, 0.12 mg; MnSO_4_⋅4H_2_O, 21 mg; CoCl_2_⋅6H_2_O, 0.30 mg; KI, 0.0780 mg.

**Table 2:** Primers used in our RT-PCR validation analysis.

Primer	Sequence (5′-3′)	Product Length (bp)	Sequence Source	GenBank Accession Number
*miR-144*-F	CGCGCGCGACAGTATAGATG	61	Database	MIMAT0001841
*miR-144*-R	AGTGCAGGGTCCGAGGTATT			MIMAT0001841
*5S rRNA*-F	CTATGCCCGATCTCGTCTGA	62	[42]	XR_007187745
*5S rRNA* -R	AGCTTACAGCACCTGGTATTCC			XR_007187745
*Nrf2*-F	GGGGAAGTCCTTGAACGGAG	115	[43]	XM_048192838
*Nrf2*-R	AACCAGCGGGAATATCTCGG			XM_048192838
*Keap1*-F	ACCAATGGGCTGAAGGAGTG	196	Database	XM_048200094
*Keap1*-R	GCACGAGGAAATCGCAACAG			XM_048200094
*NF-κB*-F	AGTCCGATCCATCCGCACTA	85	[44]	XM_048176853
*NF-κB*-R	ACTGGAGCCGGTCATTTCAG			XM_048176853
*TNF-α*-F	CGACGCTATACGGACCTTCG	232	Database	XM_048182361
*TNF-α*-R	AAGACAGGAGCCAAGGAGAAC			XM_048182361
*HO-1*-F	TGATGCCACTCAGTCCCAAG	201	Database	XM_048160979
*HO-1*-R	AGCACTTCTTTGGACCCCAC			XM_048160979
*IL-6*-F	ACGCATAGCCTACAGCGATT	140	Database	XM_048152057
*IL-6*-R	GAGCTCCAGGTCGCAATCTT			XM_048152057
*NQO1*-F	CACCACCAGTTGCGAGGAAT	95	Database	XM_048186312
*NQO1*-R	ATTCGGTCGGAGCAAAGGAC			XM_048186312
*HSP70*-F	CCCGACATGCCCTCCTTAAT	219	[44]	XM_048186826
*HSP70*-R	CACCACCCCATCTTTGGTCT			XM_048186826
*Beclin-1*-F	TCGACACATCCTTCAACGTC	163	[45]	XM_048187618
*Beclin-1*-R	ATGTATTTCCGAGCCACACC			XM_048187618
*ATG8*-F	CTCGGCTCTCAGGTGGATTC	294	Database	XM_048186054
*ATG8*-R	GCTGTGTGTGAGAGAAGCCT			XM_048186054
*P62*-F	CACTTGAGGTGCTGCTCTGA	264	Database	XM_048176526
*P62*-R	TTAACTTCGGACAGACGGGC			XM_048176526
*AKT3*-F	CGGCGAGTACAGTGTGATTG	110	Database	XM_048204587
*AKT3*-R	AGGAAGTAGCGAGGTCTCCAA			XM_048204587
*TOR*-F	TTTACACGAGCAAGTCTACGGA	180	[46]	XM_048210663
*TOR*-R	CTTCATCTTGGCTCAGCTCTCT			XM_048210663
*S6K1*-F	GGTGCATGTCACCTTATGGG	171	[46]	XM_048160409
*S6K1*-R	AGCTGGCAGCACTTCTAGTC			XM_048160409
*Fbxo25*-F	GGTGCATGTCACCTTATGGG	100	Database	XM_048178326
*Fbxo25*-R	AGCTGGCAGCACTTCTAGTC			XM_048178326
*MuRF1*-F	AGGCAGAAGAAGCAACCACT	105	Database	XM_048200485
*MuRF1*-R	GACCCGTTCGGATGTCCATT			XM_048200485
*FoxO3a*-F	TCAGGCTACTCAGGACGGAA	132	Database	XM_048190475
*FoxO3a*-R	CTGGCGTTGGAATTAGTGCG			XM_048190475
*MyoG*-F	TGGACAGCATTACAGGAACA	116	[47]	XM_048173437
*MyoG*-R	TGTTATGGTCGGTGAAAGG			XM_048173437
*MyHC1*-F	AACATGCAGACAGTGTGGCT	131	Database	XM_048175185
*MyHC1*-R	AAGCTGCTCCATGTTGGTGA			XM_048175185
*β-actin*-F	TCGTCCACCGCAAATGCTTCTA	152	Database	XM_048192430
*β-actin*-R	CCGTCACCTTCACCGTTCCAGT			XM_048192430

## Data Availability

The original contributions presented in this study are included in the article. Further inquiries can be directed to the corresponding authors.

## Supplementary Materials

The following supporting information can be downloaded at: https://www.mdpi.com/article/10.3390/antiox14101223/s1, Table S1: Effects of oxidized fish oil and *miR-144* interference on muscle texture characteristics of *M. amblycephala*.
