# Supplementary material for: The Role of miR-144/Nrf2 Pathway in Muscle Oxidative Stress Induced by Oxidized Fish Oil in Megalobrama amblycephala, with an Emphasis on Protein Oxidation
Source: Antioxidants (Basel). 2025 Oct 11;14(10):1223. doi: 10.3390/antiox14101223 (PMC12561103; doi:10.3390/antiox14101223)
Supplement: Supplementary file 1 [file antioxidants-14-01223-s001.zip › antioxidants-3888244-supplementary.pdf]

Table S1. Effects of oxidized fish oil and *miR-144* interference on muscle texture characteristics of *M. amblycephala*.

	NC	OF	OF+ago	OF+anta
Shearing force(N)	0.82±0.09	0.70±0.06	0.39±0.06	0.83±0.16
Hardness (g)	911.06±64.49	626.99±46.83	677.1±35.5	726.97±50.02
Adhesiveness (mJ)	-6.68±0.58	-5.93±0.48	-5.85±0.48	-4.56±0.32
Springiness (mm)	0.38±0.01	0.34±0.01	0.31±0.01	0.35±0.01
Cohesiveness	0.43±0.02	0.39±0.01	0.38±0.01	0.43±0.01
Gumminess (mJ)	387.83±25.51	278.57±30.58	257.58±19.45	325.22±26.53
Chewiness (mJ)	147.74±10.76	98.21±15.12	82.48±8.04	117.25±12.37
Resilience	0.3±0.02	0.25±0.02	0.23±0.02	0.29±0.01

Note: The results are expressed as the mean ± SEM, n=6.
